# Clinical Characteristics of COVID-19 Infection in Newborns and Pediatrics: A Systematic Review 

**Published:** 2020-04-18

**Authors:** Latif Panahi, Marzieh Amiri, Somaye Pouy

**Affiliations:** 1 Master Student of Nursing, Student Research Committee, School of Nursing and Midwifery, Guilan University of Medical Sciences, Rasht, Iran.; 2 Department of Emergency Medicine, Razi Hospital, School of Medicine, Guilan University of Medical Sciences, Rasht, Iran.; 3 PhD Student of Nursing, Student Research Committee, School of Nursing and Midwifery, Guilan University of Medical Sciences, Rasht, Iran.

**Keywords:** COVID-19, severe acute respiratory syndrome coronavirus 2, Child, Infant, Infant, Newborn, Coronavirus

## Abstract

**Introduction:**

World Health Organization has declared COVID-19 a pandemic and a global health emergency. Thus, it is necessary to clearly characterize clinical manifestations and management of COVID-19 infection in children to provide accurate information for healthcare workers. Accordingly, the present study was designed to review articles published on clinical manifestations and characteristics of children and infants with COVID-19.

**Methods:**

In this systematic review, medical databases including Cochrane Library, Web of Science, Embase, Scopus, SID, Medline, WHO and LitCovid were searched using English and Persian keywords including COVID-19, Pediatrics, Newborn, Coronavirus 2019, 2019-nCoV, SARS-CoV-2. Finally, data of 14 related articles were included in the study.

**Results:**

A total of 2228 children, newborns and infants were studied. Clinical manifestation in children may be mild (72%), moderate (22%) or severe (6%), and the most common symptoms include dry cough (91%) and fever (96%). According to the included articles, two children had died, one of which was a 14-year-old boy and his exposure history and underlying disease were unclear, and the other was a male newborn with gestational age of 35 weeks and 5 days, birth weight of 2200, Apgar score of 8, 8 (1 min and 5 min) and his first symptom was increased heart rate. No differences were found between male and female children regarding infection with COVID-19.

**Conclusion:**

Most pediatrics were infected with COVID-19 due to family cluster or history of close contact. Infected children have relatively milder clinical symptoms compared to infected adults. We should pay special attention to early diagnosis and early treatment in children infected with COVID-19.

## Introduction

In late December 2019, with the onset of a series of viral pneumonia and the detection of COVID-19, it spread rapidly throughout China and then the world (1-4). World Health Organization has declared COVID-19 a pandemic and a global health emergency (5-8). According to WHO reports, 196 countries and regions have been affected, 413,467 people have been infected worldwide and 18,433 have died up to March 25, 2020 (9). Iran is also one of the major countries involved with COVID-19 and according to official statistics, 23049 cases have been confirmed and 1812 people have died by March 23, 2020 (10). Coronaviruses are RNA viruses from the Coronaviridae family and Coronavirinae subfamily. The novel coronavirus that has spread worldwide after emerging in Wuhan is a beta-coronavirus (11-13). This virus has been labeled as severe acute respiratory syndrome coronavirus 2 (SARS-CoV-2) as its phylogenetic features are similar to SARS-CoV (14, 15). Infection with COVID-19 can be mild or severe and infected patients have clinical manifestation such as cough, high fever, chest pain, lethargy, weakness, muscular pain and diarrhea (16-18). Many infected children and newborns with COVID-19 have been identified all over the word. Thus, it is necessary to clearly characterize clinical manifestations and management of COVID-19 infection in this age group in order to provide accurate information for neonatologists and pediatricians. Accordingly, the present study was designed to review articles published on clinical manifestations and characteristics of children and infants with COVID-19.

## Methods

**Study design**

 The present study is a systematic review that was prepared according to the Preferred Reporting Items for Systematic Reviews and Meta-analysis (PRISMA) statement (19).

**Search process and search strategy**

**Figure 1:**
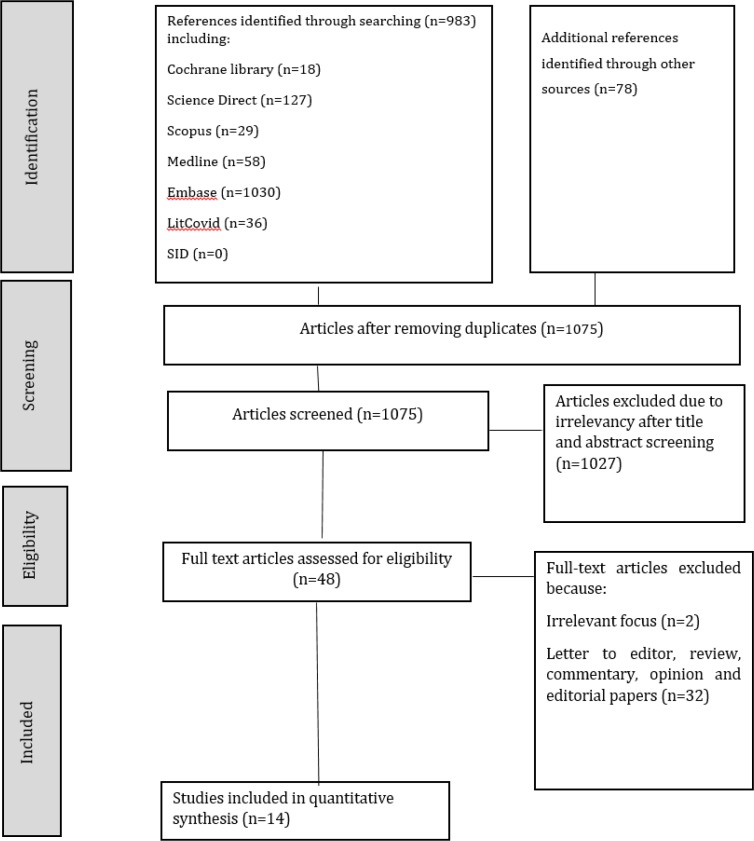
Preferred reporting items for systematic reviews and meta-analyses (PRISMA) flowchart of the present study

In this review article, a comprehensive search was conducted on medical databases including Cochrane Library, Web of Science, Science Direct, Embase, Scopus, SID, Medline, WHO and LitCovid using English and Persian keywords including COVID-19, children, newborn, child, neonate, infant, adolescent, Coronavirus 2019, 2019-nCoV, SARS-CoV-2 between 1 January and 30 March 2020 using Boolean Logics including AND, OR, NOT. We used this example syntax in searching databases: (COVID-19 OR 2019-nCoV OR Coronavirus 2019 OR SARS-CoV-2) AND (Children OR Child OR Newborn OR Infant OR Neonate OR Adolescents OR Pediatric). Initial search using these keywords yielded 983 articles that were assessed. After reviewing the titles and excluding duplicates, 60 relevant full-text articles remained. 46 of 60 articles were excluded due to lack of relevance to the purpose of the study or being a review article, letter to editor or commentary. Finally, 14 full-text articles were included. Moreover, we searched grey literature and used hand searching on references of articles that yielded 78 articles that were duplicates. Details of the search process are presented in figure 1. 

**Screening and inclusion criteria of articles**

Inclusion criteria were: All articles published as case studies, case series, cohorts or observational studies from 1 December 2019 to 30 March 2020 using specific keywords in Persian or English that dealt with characteristics of COVID-19 infection in children. Exclusion criteria were: articles published as letter to editor, review, or commentary. In order to reduce bias, data were collected by two researchers separately (SP and LP) and any disagreement was resolved through discussion with a third author (MA). Most of the articles were performed in China and there were very few studies and reports on children from USA, Italy, France or South Korea, despite the high number of infected patients with COVID-19 in those countries. Among the included studies, 1 article was from Singapore, 1 from Japan and 12 articles were from China.

**Quality assessment**

 We used the three statements of Joanna Briggs Institute critical appraisal tools in order to assess the three types of included studies (case series, case reports and retrospective cohort studies). The quality appraisal tool for case reports, case series and retrospective cohort studies have 8, 10 and 11 items, respectively. Therefore, the total score of quality assessment for articles using these checklists was 8, 10 and 11 respectively. Their answers were: yes, no, unclear or not applicable (20). The quality scores of the evaluated papers are presented in Table 1.

**Data Extraction**

We designed a data extraction form to record information, which included the name of the researchers, country, year of study, type of research, sample size, history of exposure, clinical manifestations, underlying diseases, outcome, and quality assessment score of included studies. We used descriptive statistics to summarize the etiological, demographic, and clinical characteristics of pediatric COVID-19 patients.

## Results

**Characteristics of included studies**

After searching the selected databases using MesH-matching keywords, 983 articles were obtained; furthermore, searching in grey literature and hand searching of references yielded 78 articles. After removing duplicates, 760 articles remained, 712 of which were removed due to irrelevancy to subject. Finally, 48 full-text articles were assessed and 14 full-text articles were included (fig.1) (4, 21-33). The 14 included articles consisted of case studies, case series, correspondence, commentary and letter to editor, which were published about the clinical characteristics and management of children with COVID-19. The characteristics of the studies are presented in Table 1.

**Analysis of the reports**

A total of 2228 children, newborns and infants were studied. The age range of children was 1 day to 16 years and 70.32% were boys and 29.67% were girls, 740 children (32.1%) had confirmed test of COVID-19 and 1488 (67.9%) were suspicious to COVID-19 infection. Studies have shown that children of all ages are at risk of developing COVID-19, and there is no difference between children of different genders in this regard (4). The mean duration of infection with COVID-19 to diagnosis ranged from 1 to 42 days. Clinical manifestations of COVID-19 in children have been reported as asymptomatic (92%) or with symptoms such as fever (96%), dry cough (91%), fatigue (45%) with mild upper respiratory tract symptoms (66%), abdominal pain (23%), nausea and vomiting (12%) and diarrhea (7%). But the most common manifestation of COVID-19 was reported as fever and cough. According to the included studies, the clinical types of COVID-19 in children were categorized in three domains including: mild (Upper respiratory symptoms such as pharyngeal congestion, sore throat, and fever for a short duration or asymptomatic infection, positive RT-PCR test for SARS-CoV-2, no abnormal radiographic and septic presentation), moderate (Mild pneumonia, symptoms such as fever, cough, fatigue, headache, and myalgia, no complications and manifestations related to severe conditions) and severe (Mild or moderate clinical features, plus any manifestations that suggest disease progression: Rapid breathing (≥70 breaths per min for infants aged <1 year; ≥50 breaths per min for children aged >1 year), Hypoxia, loss of consciousness, depression, coma, convulsions, Dehydration, difficulty eating, gastrointestinal dysfunction, myocardial injury, elevated liver enzymes, coagulation dysfunction, rhabdomyolysis) (12). In the surveyed children, the severity of the disease was reported as mild (72%), moderate (22%) and severe (6%). Most of the children (78%) were asymptomatic and only 5% of asymptomatic children developed clinical symptoms such as dyspnea or hypoxia, and 0.6% developed acute respiratory distress syndrome (ARDS) or multi-organ failure. Also, all articles stated that, based on existing knowledge, the childhood illness is much less severe than that of adults and its cause is unknown. In this regard, we assessed underlying diseases of children, but most of them did not have any underlying disease (42.8%) and some studies did not state if underlying illnesses were present or not (57%). Studies have also shown that lower respiratory tract involvement is rarely seen in children and most of them have upper respiratory tract involvement. Most of the reported cases had improved within 1 to 2 weeks and were discharged from the hospital (99.72%); however, some remained under treatment (0.13%) or had died (0.089%). According to articles, two children had died. One of them was a 14-year-old boy who lived in Hubei province, but no information was provided about his exposure history, underlying disease or comorbidity. The other case was a male newborn with gestational age of 35 weeks and 5 days, birth weight of 2200, and Apgar score of 8, 8 (1 min and 5 min) and his symptoms included increased heart rate (first symptom), refractory shock and gastric bleeding. Other complications included multiple organ failure and disseminated intravascular coagulation (DIC). This newborn’s mother was 30 years old and had undergone Cesarean section and her first symptom was fever, which began 3 days after delivery. Her umbilical cord, placenta and amniotic fluid were normal. No premature rupture of membranes or intrauterine fetal distress was reported. These finding indicate that the mortality rates in children are very low. Researchers stated that most of the children had been infected with COVID-19 through family cluster infection and close contact with infected people (98.69%); some were infected through travel to epidemic center of COVID-19 (0.089%) or due to an unknown origin (0.26%). In the studied children, for assessing COVID-19 infection, samples were taken from nasopharyngeal secretions in 2208 cases (99.10%), from blood in 2144 cases (96.22%), from stool in 3 cases (0.13%), from saliva in 1 case (0.044%), and from oropharyngeal stations in 19 cases (0.85%). It was notable that in the 3 cases that stool samples were assessed for COVID-19 infection, the result was positive 11-17 days (mean=15day) after symptom onset. So, one of the major concerns is the transmission of COVID-19 in children through fecal–oral route, because viral shedding has been found to take about 2-3 weeks after clinical manifestation of disease. Therefore, fecal–oral transmission should be considered in addition to droplets in transmission of COVID-19 in children and infants. Studies, including predictors of disease severity, have indicated that children with younger age, underlying diseases, and immunodeficiency diseases are at higher risk for severe infection. So, infants and preschool-aged children are at greater risk of developing severe forms of the disease compared to older children. Also, one of the most important methods of screening children with COVID-19 is using exposure history and clinical symptoms, and unlike adults, chest CT scan findings are not valid in children.

**Table 1:** Characteristics of included studies

Study	Gender/Age	No	Cause	Onset	UD	Severity of disease (%)	Clinical manifestations	ST	QS	Outcome	Key Findings
Dong et al. (2020) (4) China	Boy (56.6%) Girl (44.4%)/ Median: 7 years	2143	Exposed to a COVID-19 case or lived in an epidemic area	2 days (Range: 0-42 days)	ND	Asymptomatic: 4.4 Mild: 50.9 moderate: 38.8	Fever, respiratory symptoms or digestive symptoms, fatigue	Cohort Study	7	Recovered:2142 Died: 1	Children at all ages appeared susceptible to COVID-19. Clinical manifestations of children’s COVID-19 cases were less severe than adult patients.
Ji et al. (2020) (23) China	Two boys: 9 and 15 years-old	2	Travel history to COVID-19 center	1-2 days	No	Mild: 100	Fever, pharyngeal congestion, mild diarrhea	Case Series	8	Recovered: 2	Infected children have relatively milder clinical symptoms than infected adults.
Yong Park et al. (2020) (28) Japan	One 10 year-old girl	1	Contact with infected family member	13 days	ND	Mild: 100	Fever	Case report	8	Recovered: 1	Children are less infected and less ill with COVID-19.
Zhu et al. (2020) (33) China	Boy (80%) Girl (20%)/ One day-old newborn	10	Born from infected mother with COVID-19 and close contact after birth	1-9 days	ND	Mild: 100	Fever, shortness of breath, thrombocytopenia, rapid heart rate, vomiting	Case series	8	Recovered:9 Died: 1	2019-nCoV infection may have adverse effects on newborns, causing problems such as fetal distress, premature labor, respiratory distress and even death.
Li et al. (2020) (25) China	One boy and one girl	2	Contact with infected family member	3-10 days	ND	Mild :100	Cough, runny nose	Case report	8	Not discharged yet: 2	Infection with COVID-19 is milder in children and recovery is faster.
Liu et al. (2020) (26) China	2 female/ 2 male: 2 & 11 month, 5 &9 years	4	Exposure history	ND	ND	Mild: 100	Fever, Cough, Fatigue	Cohort	9	Recovered: 4	Exposure history and clinical symptoms were more helpful for screening in children versus chest CT.
Cui et al. (2020) (22) China	A 55 days-old female infant	1	Exposure to her infected parents and family	17 days	No	Severe:100	Rhinorrhea, dry cough	Case report	9	Recovered: 1	This case study highlights that children with COVID-19 can also present with multiple organ damage and rapid disease changes like adults.
Wang et al. (2020) (31) China	A 36 hours-old male	1	Born from infected mother with COVID-19 and close contact after birth	Immediately after birth	No	Mild: 100	Vomiting	Case report	8	Recovered: 1	Vertical transmission of SARS-CoV-2 through placenta and its short-term and long-term harm to offsprings is still unclear.
Qiu et al. (2020) (12) China	Female: 46%; Male: 64%/ Age: 1-16 years	36	Close contact with family members and history of exposure to the epidemic area	ND	ND	Mild: 47 Moderate: 53	Fever, dry Cough	Cohort Study	9	Recovered: 36	The high rate of asymptomatic children with COVID-19 indicates the difficulty of identifying pediatric patients
Kam et al. (2020) (34) Singapore	A 6 months-old boy	1	Close contact with family members	23 days	No	Mild: 100	Asymptomatic	Brief report	8	Not discharged yet: 1	Infants can be asymptomatic despite high viral load
Zhou et al. (2020) (35) China	Female: 66.6% Male: 33.3%/ Median: 1-7 years	6	Unknown	2-13 days	No	Mild: 33.3 Moderate: 50 Severe: 16.6	Fever, dry Cough, vomiting	Case report	8	Recovered: 6	Covid-19 in children can cause moderate-to-severe respiratory illness
Jiehao et al. (2020)(36) China	Female: 70% Male: 30%/ Median: 6 years	10	Close contact with family members and exposure to the epidemic area	2-10 days	ND	Mild: 40 Moderate: 60	Fever, dry Cough, sore throat, stuffy nose, sneezing	Case series	8	Recovered: 10	Children with COVID-19 usually present with milder respiratory infections, compared to adult cases
Zhang et al. (2020) (37) China	Female: 70% Male:30%/ Median: 6 years	10	Born from infected mother with COVID-19	Immediately after birth	No	Mild: 40 Moderate: 60	Fever, vomiting	Case series	7	Recovered: 10	Timely termination of pregnancy will not increase the risk of premature birth and newborn asphyxia
Tang et al. (2020) (38) China	A 10 year-old boy	1	Close contact with infected COVID-19 case	17 days	ND	Mild: 100	Asymptomatic	Case report	7	Recovered: 1	Stool of COVID-19 patients might serve as a vehicle for virus transmission

ND: Not Determined; UD: underlying disease; QS: Quality score; ST: study type.

## Discussion

The present study surveyed the published literature on the clinical characteristics of COVID-19 in infants and children. A literature review of studies demonstrated that children with COVID-19 can be completely asymptomatic or have mild to moderate symptoms that can result in not being diagnosed (undocumented). Findings of Ogimi et al. showed that younger age, especially in children younger than school age, underlying diseases and immunosuppressive diseases are predictors of disease severity (39). In one study, Li et al. indicated that about 86% of early infections with COVID-19 in patients in China were undiagnosed (40). Despite the low risk of infection transmission by undiagnosed cases, they are responsible for 79% of early infections (41). So, this raises concern about asymptomatic children that are taken care of by adults and elderly people, as they can be a source of COVID-19 transmission. According to studies, children make up 2% of COVID-19 cases in China, 1.2% in Italy and 5% in US (9, 42, 43). These statistics are in line with those of SARS epidemic, where only 6.9% of infected patients were children (41).

According to early reports, presence of comorbidities including diabetes, hypertension, chronic respiratory disease and cardiac disease is a risk factor for poor prognosis in the adult population. Studies showed that 67.2% of patients who died from COVID-19 had a comorbidity (44). In our review article, we presented clinical features of children and their comorbidities (if present), but until now, no article has been published on the correlation between comorbidities and disease outcome in children. Clinical manifestations of COVID-19 that have been reported in children and infants include fever, dry cough, fatigue, symptoms of upper respiratory tract infection such as runny nose, and gastrointestinal symptoms such as anorexia, diarrhea, nausea and vomiting. The most common symptoms that have been reported are fever and dry cough. Unlike adults, inferior respiratory tract involvement rarely occurs in children (17, 18, 22, 33). Study of literature indicated that children of all ages were susceptible to COVID-19, but were less likely than adults to develop symptoms (4, 20, 22).

The underlying cause of the lower incidence and milder manifestations of COVID-19 in children is obvious. Children, especially younger children, have been infected with numerous viral infections. It is possible that repetitive exposure to numerous viruses can boost the immune system against SARS-CoV-2 infection. Additionally, studies have shown that SARS-CoV-2 tends to attach to angiotensin-converting enzyme (ACE), which is premature in children and this may result in a low rate of infection with SARS-CoV-2 (41, 42).

It is very remarkable that various published articles reported high rates of lymphocytopenia. A study by Guan et al., which was performed on 1099 patients with COVID-19, showed that 82.3% of them had lymphocytopenia (45). While, in a study on 171 infected children, only 3.5% showed lymphocytopenia and in a study by Henry et al. only 3% of children had lymphocytopenia (46). So, further studies are needed to assess the role of lymphocytes and severity of COVID-19 in children. 

Various studies have also found that most children have been infected with COVID-19 due to family cluster transmission or close contact with the infected patient (20, 23). The study found that viral-shedding occurs more than 4 weeks after the onset of the disease, raising major concerns about fecal-oral transmission of COVID-19 in children. Fecal-oral transmission is especially very important in children who have not yet received toilet training, which is very important for infants and preschool aged children. Therefore, parents need to be well educated in this regard(47).

 A study by Dong et al. showed that gender had no effect on the incidence or severity of COVID-19(4). In addition, vertical intrauterine transmission from pregnant mothers to newborns has not been reported yet. According to studies, samples taken from cord blood and placenta of pregnant women with positive COVID-19 had negative results and one study reported that 30 newborns of COVID-19 infected mothers had negative test results. Also, it should be noted that most of these neonates, were born via Caesarean section (40, 48, 49). 

According to the findings of Liu et al., paying attention to clinical symptoms and history of exposure to COVID-19 have high diagnostic value in children, while chest CT scan is not able to accurately detect the severity of the disease in children(28). In conclusion, the results of this study indicate that paying attention to children's health is crucial during the COVID-19 pandemic and that effective training should be given to parents.

## Limitations

One of the important limitations of our study was that we could not use Chinese databases and journals. Also, we could not study the full-text of some of the Chinese articles and we had to just rely on English-language summaries.
